# Advanced Sensors Technology in Education

**DOI:** 10.3390/s19194155

**Published:** 2019-09-25

**Authors:** Rubén González Crespo, Daniel Burgos

**Affiliations:** 1 Faculty of Engineering, Universidad Internacional de La Rioja, Av. de la Paz 137, 26006 Logroño, La Rioja, Spain; 2 Research Institute for Innovation & Technology in Education (UNIR iTED), Universidad Internacional de La Rioja (UNIR), 26006 Logroño, La Rioja, Spain

**Keywords:** educational technologies, ambient assisted living (AAL), augmented reality, signal processing, computing devices

## Abstract

The topic presented will show how different kinds of sensors can help to improve our skills in learning environments. When we open the mind and let it take the control to be creative, we can think how a martial art would be improved with registered sensors, or how a person may dance with machines to improve their technique, or how you may improve your soccer kick for a penalties round. The use of sensors seems easy to imagine in these examples, but their use is not limited to these types of learning environments. Using depth cameras to detect patterns in oral presentations, or improving the assessment of agility through low cost-sensors with multimodal learning analytics, or using computing devices as sensors to measure their impact on primary and secondary students’ performances are the focus of this study as well. We hope readers will find original ideas that allow them to improve and advance in their own researches.

## 1. Introduction

User tracking becomes the key to personalised learning and teaching. To provide adaptive counseling to the student and the lecturer, an automatic system must collect, categorise and process a large amount of data under a number of rules. The more collected data there is, the more accurate is the recommendation. Further, the more open the system is, the bigger the data set becomes. We find, therefore, three main features to design and produce a smart approach to current education: User tracking; big data; open access.

Indeed, one of the most well-known requirements in educational settings is the need to know what happens during a course, lesson plan or full academic programme. Learning Analytics can then be applied to cluster, depict and process data to support a number of objectives. This is true for any type of education but for open education in particular, which has multiple dimensions of openness. Educators (i.e., teachers, professors, tutors, etc.) and practitioners of open education need to reshape the course plan according to the actual features of the learners (e.g., learning styles, motivation, performance, etc.), on the one hand, and they, therefore, require real-time analytical information to supervise, assess, adapt and offer feedback to the learners. Conversely, open education offers specific opportunities through online learning using open educational resources (OER). The online environments and platforms provide a huge amount of data on every activity. More importantly, open education with open teaching and learning currently is commonly shaped by a learner-centred approach that pushes the learners to be the driver of their own learning. That is, learners require awareness to self-assess their progress along the course and make decisions regarding their next steps.

Sensors become the best ally, in this context, to improve the learner’s performance and to boost the teacher’s didactics. Thanks to a large collection of past and accurate data, the researcher can design models, tools and frameworks to support those objectives. These sensors can be either physical, like with wearables, biometrics and assorted hardware, or virtual, like triggers in games, virtual reality spaces or augmented reality scenarios. Both types of sensors can be applied to a wide range of educational contexts: Used in a Learning Management System (LMS) or a Content Management System that processes all the user data collected; in a session to provide the most adapted recommendations to the user; in a training court where hardware collects physical moves and evaluates the players or the dancers; in real-time presentations so the oral language and body language skills are shaped and improved; in working with Lego pieces to learn programming; in using game-based learning; in calligraphy training to support soft skills; using eyewear to improve specific procedures like Cardiopulmonary Resuscitation (CPR) and in supporting specific cognitive accessible time displays. Indeed, sensors are the most accurate resource to measure the most subtle user interaction and beyond—something the user is not aware of and, yet, affects their behavior and future decisions.

## 2. Contributions

Baldominos et al. [1] proposes to evaluate two aspects of such system focused on children: (1) the influence of the order of recommendations on user exploratory behavior; (2) the impact of the choice of the recommendation algorithm on engagement. The assessment requires the analysis of the number of clicks performed according to the recommendations, depending on their order, and an A/B/C testing where two standard recommendation algorithms are compared with a random recommendation that serves as a baseline. The results suggest a direct connection between the order of the recommendation and the interest raised, and the superiority of recommendations based on popularity compared to other alternatives.

Hasegawa et al. discusses the importance of typing skills in the digital information society of this generation [2]. As a method to improve typing speed, the authors focus on the training of touch-typing that enables typing a key without looking at the keyboard. To support touch-typing training, it is efficient to apply a penalty if a learner looks at the keyboard; however, to realize the penalty method, the computer needs to be able to recognize whether the learner looked at the keyboard. Hasegawa et al. propose a method to detect a learner’s eye gaze, specifically, by using sensor eyewear to detect whether the learner looks at the keyboard and then evaluating the detection accuracy of the proposed method.

In the third paper [3], a device to train children in time orientation is designed, developed and evaluated. It is framed within a long-term cooperation agreement between a university and a special education school. It uses a specific cognitive accessible time display. Time left in the day is represented by a row of initially lit luminous elements. Time passing is represented by turning off, sequentially and gradually, each luminous element every 15 minutes. An agenda is displayed relating time to tasks, with standard pictograms for further accessibility. Notifications of tasks-to-come, both for management support and anticipation of changes, uses visual and auditory information Finally, the agenda is described in an Alternative and Augmentative Communication pictogram language already used by the children, supporting individual and class activities on the agenda.

Naranjo et al. introduces CloudTrail-Tracker, an open-source platform to obtain enhanced usage analytics from a shared AWS account [4]. The tool provides the instructor with a visual dashboard that depicts the aggregated usage of resources by all the students during a certain time-frame, and the exclusive use of AWS for a specific student. To facilitate self-regulation of students, the dashboard also depicts the percentage of progress for each lab session and the pending actions by the student. The dashboard has been initiated for four Cloud subjects who use different learning methodologies (from face-to-face to online learning), and the students have highlighted the usefulness of the tool for Cloud instruction in AWS positively. This automated procurement of evidence of student activity on the Cloud results in close to real-time learning analytics, useful both for semi-automated assessment and student self-awareness of their own training progress.

Di Mitri et al. investigate to what extent multimodal data could be used to detect mistakes during Cardiopulmonary Resuscitation (CPR) training [5]. They complement the Laerdal QCPR ResusciAnne mannequin with the Multimodal Tutor for CPR, a multi-sensor system consisting of a Microsoft Kinect for tracking body position and a Myo armband for collecting electromyogram information. The results of the experiment show that multimodal data can provide accurate mistake detection as compared to the ResusciAnne mannequin baseline. They also show that the Multimodal Tutor for CPR can detect additional CPR training mistakes, such as the correct use of arms and body weight. Finally, to investigate user feedback in the future implementations of the Multimodal Tutor for CPR, they conduct a questionnaire to collect valuable feedback aspects regarding the CPR training.

The sixth paper from Fernández-Soriano et al., aims to study the relationship between the type of computing device from which students access the LMS and how it affects their performance [6]. To achieve this, the LMS accesses students in a school comprising elementary to bachelor’s degree stages who are monitored by means of different computing devices acting as sensors to gather data, such as the type of device and operating system used by the students. The main conclusion is that students who access the LMS significantly improve their performance and, additionally, the type of device and the operating system has an influence on the number of passed subjects. Moreover, a predictive model is generated to predict the number of passed subjects according to these factors, showing promising results.

Limbu et al. presents a pilot study done with the calligraphy trainer to evaluate the mental effort required by various types of feedback provided by the application [7]. The participants use the application to learn three characters from the Devanagari script. The results show higher mental effort in the treatment group when all types of feedback are provided simultaneously. The mental efforts for individual feedback are similar to the control group. To conclude, the feedback provided by the calligraphy trainer does not impose greater mental effort and, therefore, the design considerations of the calligraphy trainer can be insightful for multimodal feedback designers.

Students from different countries are able to adapt their learning material by programming and designing games for their academic subjects, therefore integrating the game mechanics, dynamics, and aesthetics into the academic curriculum. Gaeta et al. [8] focuses on presenting the validation context as well as the evaluation tools developed. The Hassenzahl model and AttrakDiff survey are used to measure users’ experience and satisfaction and to understand emotional responses. Following two years using code-making apps, the pupils process knowledge from their academic subjects spontaneously as game-based embedded knowledge. The students demonstrate creativity, a practical approach, and enthusiasm regarding making games focused on academic content that leads them to learning, using mobile devices, sensors, images, and contextual information.

Cornide-Reyes et al. presents an exploratory study that analyzes the collaboration and communication of students in a Software Engineering course who perform a learning activity simulating Scrum with Lego® bricks [9]. Data from the Scrum process is captured and multidirectional microphones are used in the retrospective ceremonies. Social network analysis techniques are applied and a correlational analysis is carried out with all the registered information. The results allow for the detection of important relationships and characteristics of the collaborative and non-collaborative groups, with productivity, effort, and predominant personality styles in the groups. The authors can conclude that Multimodal Learning Analysis techniques offer considerable feasibility to support the process of skills development in students.

In paper [10], Schneider et al. show how a multimodal sensor-based application designed to support the development of public speaking skills can be extended modularly with a Virtual Reality real-time feedback module (VR module), which makes using it more immersive and comprehensiv. The study consists of a formative evaluation and has two main objectives: (1) a technical objective is concerned with the feasibility of extending the multimodal sensor-based application with an immersive VR Module; (2) a user experience objective focuses on the level of satisfaction of interacting with the VR extended by the multimodal sensor-based application. Results from their test show the feasibility of modularly extending existing multimodal sensor-based applications and, in terms of learning and user experience, results indicate a positive attitude of the participants toward using the application.

Speaking and presenting in public are critical skills for academic and professional development. In paper [11], Roque et al. collect data from 222 Computer Engineering (CE) undergraduate students at three different times, over two different years. Regarding each presentation, using a developed system and Microsoft Kinect, they detect 12 features related to corporal postures and oral speaking. These features are used as input for the clustering and statistical analysis that allows identification of three different clusters in the presentations of both years. A Wilcoxon rank-sum test allows them to evaluate the evolution of the presentation attributes over each year and point out a convergence in terms of the reduction of the number of features statistically different between presentations given at the same course time. The results can help to give students automatic feedback in terms of their postures and speech throughout the presentations and may serve as baseline information for future comparisons with presentations from students coming from different undergraduate courses.

The aim of the paper [12], is to analyze how the modification of the pitch size in small-sided games (SSGs) affects the physical demands of the goalkeepers. The data gathered is used to compute players’ spatial exploration index, their standard ellipse area, and their predictive ellipse area. Additionally, the distance covered, distance covered in different intensities and accelerations/decelerations are used to assess the players’ physical performance. The results show differences between small and large SSGs in relation to the distances covered at different intensities and pitch exploration. Additionally, intensities are lower when the pitch size is larger and the pitch exploration variables increase along with the increment of the pitch size.

Dancing, which consists of feeling the music and expressing it in rhythmic movements of the body, is an activity that positively enhances the mood of people. In paper [13], Romano et al. present the first implementation of the Dancing Coach (DC), a generic system designed to support the practice of dancing steps which, in its current state, supports the practice of basic salsa dancing steps. Results from the user test show that participants stated they had learned the basic salsa dancing steps, allowing them to move to the beat and use body coordination in a fun way. Results also point out some direction on how to improve the future versions of the DC.

To leverage engagement in the learning of the physics STEM area, teachers try to come up with creative ideas about the design of their classroom lessons. Sports-related activities can foster intuitive knowledge about physics. In the paper [14], Corbi et al. begin by reporting a user study among high-school students showing that the physics concept regarding the moment of inertia can be understood by watching live exhibitions of specific aikido techniques. Based on these findings, they later present Phy + Aik, a tool for educators that enables the production of innovative visual educational material consisting of high-quality videos (and live demonstrations) synchronized/tagged with the inertial data collected by sensors and visual tracking devices. Authors think that a similar approach, where sensors are automatically registered within an intelligent framework, can be explored to teach other difficult-to-learn STEM concepts.
